# Tau seeding in cases of multiple sclerosis

**DOI:** 10.1186/s40478-022-01444-2

**Published:** 2022-10-11

**Authors:** Michael S. LaCroix, Hilda Mirbaha, Ping Shang, Stephanie Zandee, Chan Foong, Alexandre Prat, Charles L. White, Olaf Stuve, Marc I. Diamond

**Affiliations:** 1 Center for Alzheimer’s and Neurodegenerative Diseases, Peter O’Donnell Jr. Brain Institute, NL10.120, University of Texas Southwestern Medical Center, 6000 Harry Hines Blvd., Dallas, TX 75390 USA; 2 Department of Pathology, UT Southwestern Medical Center, Dallas, TX USA; 3 Centre de Recherche du Centre Hospitalier de l’Université de Montréal (CRCHUM), Neuroimmunology Research Laboratory, Montreal, Quebec H2X 0A9 Canada; 4 Department of Neurology, UT Southwestern Medical Center, Dallas, TX USA; 5 Neurology Section, VA North Texas Health Care System, Dallas, TX USA; 6 Department of Neurosciences, Faculty of Medicine, Université de Montreal, Montreal, Quebec H3T 1J4 Canada

**Keywords:** Multiple sclerosis, Tauopathy, FRET biosensor, Tau seeding activity, tau, prion, propagation, neurodegeneration

## Abstract

Relapsing remitting multiple sclerosis (MS) is an inflammatory demyelinating disorder of the central nervous system that in many cases leads to progressive MS, a neurodegenerative disease. Progressive MS is untreatable and relentless, and its cause is unknown. Prior studies of MS have documented neuronal accumulation of phosphorylated tau protein, which characterizes another heterogeneous group of neurogenerative disorders, the tauopathies. Known causes of tauopathy are myriad, and include point mutations within the tau gene, amyloid beta accumulation, repeated head trauma, and viral infection. We and others have proposed that tau has essential features of a prion. It forms intracellular assemblies that can exit a cell, enter a secondary cell, and serve as templates for their own replication in a process termed “seeding.” We have previously developed specialized “biosensor” cell systems to detect and quantify tau seeds in brain tissues. We hypothesized that progressive MS is a tauopathy, potentially triggered by inflammation. We tested for and detected tau seeding in frozen brain tissue of 6/8 subjects with multiple sclerosis. We then evaluated multiple brain regions from a single subject for whom we had detailed clinical history. We observed seeding outside of MS plaques that was enriched by immunopurification with two anti-tau antibodies (HJ8.5 and MD3.1). Immunohistochemistry with AT8 and MD3.1 confirmed prior reports of tau accumulation in MS. Although larger studies are required, our data suggest that progressive MS may be considered a secondary tauopathy.

**Supplementary Information:**

The online version contains supplementary material available at 10.1186/s40478-022-01444-2.

## Introduction

Multiple sclerosis (MS) is an inflammatory demyelinating disorder of the central nervous system that in many cases leads to neurodegeneration [1]. MS most commonly presents with a relapsing–remitting phenotype (RRMS), in which clinical findings often coincide with magnetic resonance imaging (MRI) abnormalities. RRMS responds to a variety of immunosuppressive treatments [2, 3]. However, a substantial fraction of RRMS patients transition to a secondary-progressive neurodegenerative phase that lacks signs of acute inflammation, and does not respond to immunosuppression. A minority of cases present with a chronic progressive course at the outset [2, 3]. Epidemiological evidence supports a primary neurodegenerative process shared in all cases of MS [4, 5]. The cause of progressive MS is unknown, but prior reports have documented accumulation of phosphorylated forms of the microtubule-associated protein tau [6, 7].

**Fig. 1:**
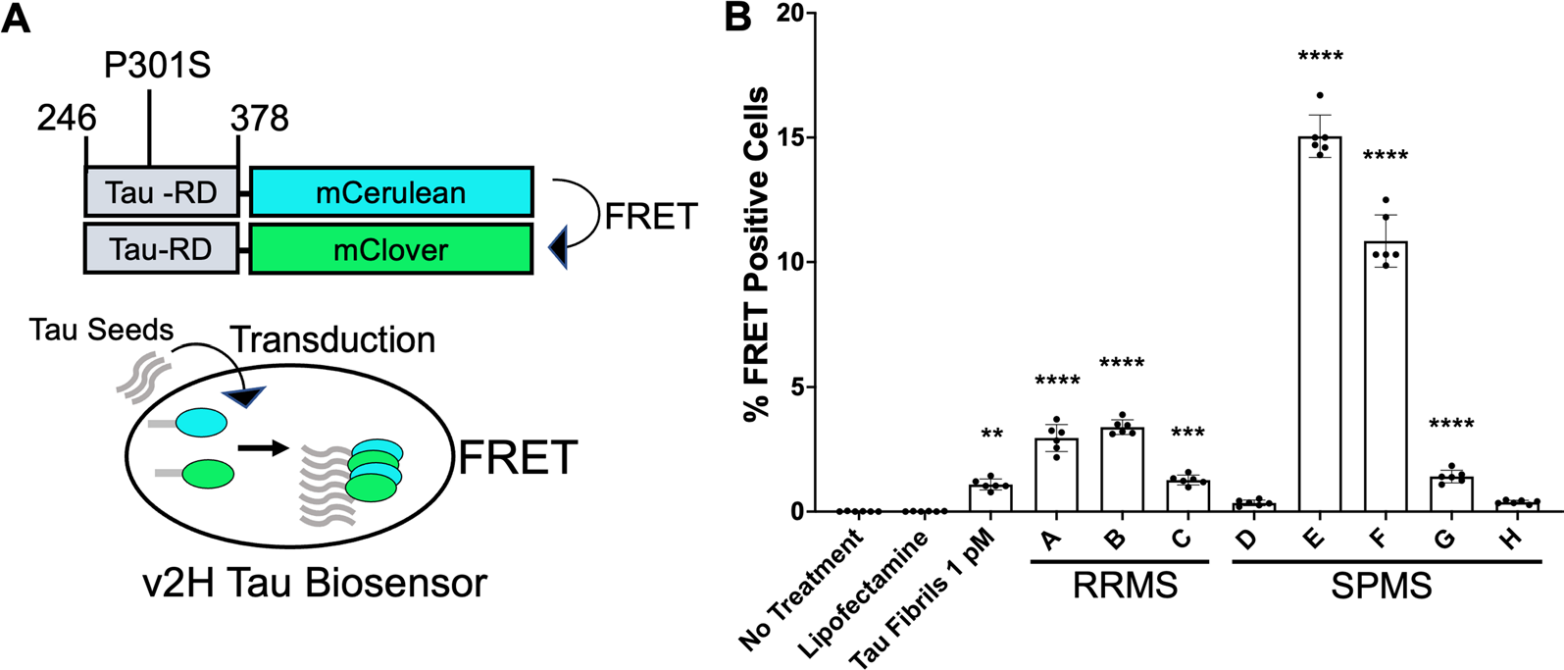
Tau biosensors detect tau seeds in homogenates from MS brains. **A** Schematic of the FRET-based tau biosensor cell assay for the detection of tau seeds. HEK293 cells express tau repeat-domain with mCerulean and mClover tags that produce FRET upon close association. Lipofectamine-mediated transduction of exogenous tau seeds initiates aggregation of tau-RD constructs, which is quantified via FRET flow cytometry. **B** Seeding was detected in 6/8 MS brains with either RRMS (**A**–**C**) or SPMS (**D–H**) based on immunopurification of tau seeds using MD3.1 antibody. Columns represent the mean % FRET positive cells of six technical replicates (dots). Statistical significance was determined by performing one-way ANOVA followed by Dunnett’s multiple comparisons testing of all samples compared against Lipofectamine treated negative controls, **p < 0.01, ****p < 0.0001. Errors bars = S.D

The intracellular accumulation of tau assemblies, or aggregates, underlies myriad disorders collectively known as “tauopathies” [8]. Many tauopathies feature detergent-insoluble filaments, which are composed of distinct, disease-associated structures [9, 10]. Considerable experimental evidence indicates that prion mechanisms underlie the progression of neurodegenerative tauopathies, whereby pathological assemblies that form in one cell exit to gain entry to connected neurons, and thereby propagate disease through specific brain networks in a process termed “seeding” [11]. Unique tau assembly structures, termed “strains,” propagate in vivo by serving as templates for their own replication, dictate rates of progression and neuronal vulnerability in mouse models, and thus likely account for phenotypic variability in humans [9, 12]. Seed detection has been facilitated by the development of specialized “biosensor” cell lines that express the tau repeat domain (which forms the core of amyloid assemblies) containing a single disease-associated mutation (P301S) fused to fluorescent proteins (Fig. 1A). When tau seeds enter biosensor cells, they initiate aggregation of tau, which is then quantified via flow cytometry based on fluorescence resonance energy transfer (FRET). Biosensor cells are highly sensitive and specific for tau pathology [13, 14], and have been used by our group and many others to quantify levels of pathological tau in a variety of disease states [15–18]. Recently, we have augmented the sensitivity of detection through development of conformation-specific antibodies that preferentially bind seed-competent forms of tau—revealing seeding otherwise undetectable by detergent fractionation or phospho-tau antibody staining [19, 20]. MD3.1, which was created by mimicking a neoepitope from seed-competent tau monomer [21], preferentially binds tau seeds after immunoprecipitation or immunostaining of tauopathy brain [19].

Several studies have described tau pathology in progressive MS, as measured by immunohistochemistry to detect phospho-tau [6, 7, 22]. Insoluble tau has been reported only in progressive MS, but not in relapsing MS. These findings may reflect the cause of neurodegeneration [6, 7], however no prior studies have evaluated MS brain for the presence of tau seeds. We hypothesized that if tau mediates neurodegeneration in MS, seeds should be present.

To address this question, we initially analyzed brain homogenates from 8 MS subjects, enriching for seeding by immunoprecipitation with MD3.1. We detected seeds in 6/8 cases (Fig. 1B). Information about the subjects and the regional source of samples is available (Additional file 1: Table 1). Both forms of MS had detectable seeds: 3/3 cases of RRMS, and 3/5 cases of SPMS.

**Fig. 2:**
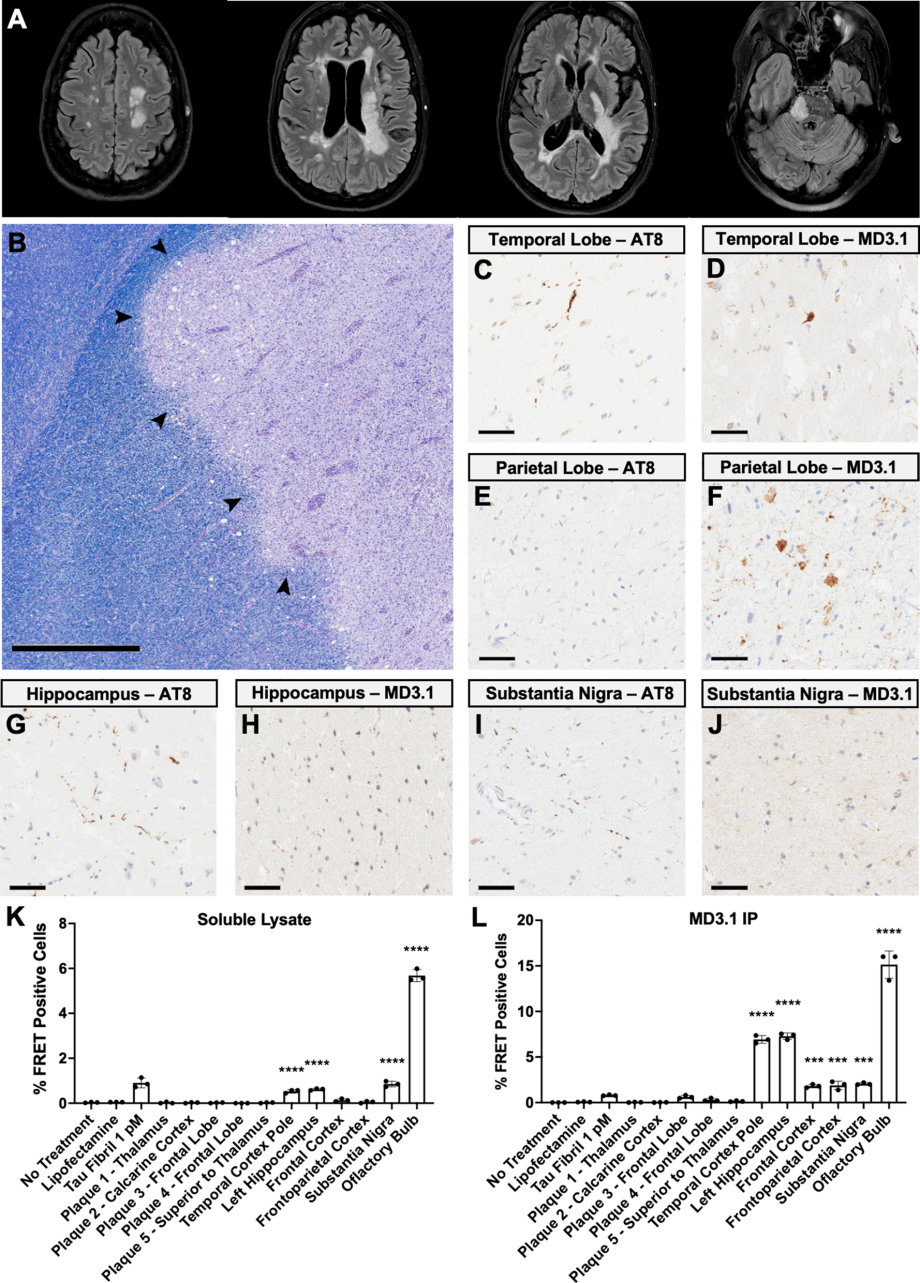
Anatomic distribution of tau seeding in an MS subject. The brain of an MS subject was preserved frozen, and then dissected to the indicated regions. Indicated regions were fixed for immunohistochemistry. For the tau seeding assay, unfixed tissue was homogenized to create total clarified lysate [10% (wt/vol)] followed by immunoprecipitation with MD3.1 to enrich for tau seeds. **A** Axial FLAIR MRI antemortem images showed extensive demyelination. **B** Periphery of an MS plaque was stained with Luxol fast blue-PAS-hematoxylin, showing preserved myelin in adjacent brain (left) and loss of myelin within the plaque (right). The plaque also contained abundant macrophages, and the interface (indicated with arrowheads) between plaque and adjacent brain contained many swollen axons. Scale bar = 1 mm. (C-J) Tau immunohistochemistry (AT8 and MD3.1) in plaque-adjacent brain regions. Scale bars = 50 µm. **C**, **D** temporal lobe, showing a collection of AT8-immunoreactive neuropil threads and MD3.1-immunoreactive tangle-like structures in 2 neurons; **E**, **F** parietal lobe, showing MD3.1-immunoreactive structures at the periphery of a plaque (consistent with swollen axons), but no AT8 immunoreactivity; **G**, **H** hippocampus, showing AT8-immunoreactive neuropil threads in entorhinal cortex, which are MD3.1-negative; and **I**, **J** substantia nigra, showing sparse AT8-immunoreactive neuropil threads, but no focal MD3.1 immunoreactivity. **K** Tau seeding in total clarified lysate from various regions of an MS brain. **L** Tau seeding in pellets after immunoprecipitation with MD3.1. Columns represent the mean % FRET positive cells from three technical replicates (dots). Statistical significance was determined by performing one-way ANOVA followed by Dunnett’s multiple comparisons testing of all samples vs. Lipofectamine treated negative controls, *** = p < 0.001, **** = p < 0.0001. Errors bars = S.D

To better understand the relationship between MS plaques and tau seeding, we prepared homogenates from plaque-bearing and adjacent brain tissues from a deceased 52 year old female subject with a 19 year history of RRMS that was well controlled until a rapid decline. Axial FLAIR MRI images indicated abnormalities in the periventricular white matter, corpus callosum, cortical gray matter, and brainstem (Fig. 2A).

Upon neuropathological examination, we observed characteristic findings of active phase demyelinated plaque formation in plaque-bearing regions (Fig. 2B). We also observed evidence of pathological tau accumulation based on immunoreactivity with anti-tau monoclonal antibodies AT8 and MD3.1 (Figs. 2C–J).

We next tested soluble brain homogenates for seeding using tau biosensor cells. We detected no seeding in homogenates from plaque-bearing tissue. By contrast, we easily detected seeding from soluble homogenates in neighboring regions, including the temporal cortex, hippocampus, substantia nigra and the olfactory bulb (Fig. 2K).

We also performed immunoprecipitation from brain homogenates, and tested the resulting pellets for seeding. We detected signal in regions we had previously observed. We also detected seeding in the frontal and parietal cortices (Fig. 2L).

In summary, we detected tau seeding activity and evidence of pathological tau accumulation in tissues adjacent to MS plaques, whereas the plaques themselves had no seeding activity and minimal evidence of phospho-tau accumulation. This could be because the neuronal content of plaques was reduced due to gliosis. MD3.1 efficiently enriched tau seeds from plaque-adjacent regions and detected disease-associated tau accumulation based on immunohistochemistry. Our findings in this regard are consistent with prior reports of tau accumulation in MS brain [6, 7, 22].

Distinct tau strains are associated with different tauopathies [9, 10], and create unique patterns of transmissible pathology upon inoculation into experimental mouse models [12]. Anti-tau antibodies directed against distinct epitopes, especially within the repeat domain, differentially bind different seed conformers [20]. We tested a panel of antibodies for their ability to immunoprecipitate tau seeds (Additional file 2: Fig. S1A). The overall pattern of tau immunoprecipitation efficiency from MS was relatively similar to AD, but one monoclonal antibody (MD6.1) failed to bind seeds of the MS brain homogenate, while it did so for AD (Additional file 2: Fig. S1B–D). This could indicate that tau seeds in MS have a distinct conformation, but this will require further study.

The cause of progressive MS is unknown, and in contrast to RRMS, which is highly responsive to immunosuppression, there is no effective treatment. In tauopathies, transcellular propagation of pathology mediated by tau seeds has been proposed as a cause of disease progression. Thus, our finding of tau seeds in MS points to tau as a mediator of neurodegeneration, presumably generated by inflammation. This has potentially important therapeutic implications. It is unknown whether chronic inflammation directly causes tau pathology, but it is remarkable that tau deposits have been described in association with other inflammatory CNS diseases such as post-encephalitic Parkinsonism, Nodding syndrome, chronic traumatic encephalopathy, and subacute sclerosing panencephalitis [23–27]. Future studies will be required to test more definitively a causal relationship between inflammation and tauopathy.

### Methods

#### Biosensor cell line

Sensitive second-generation tau biosensor cells termed v2H [13] were used for seeding assays. These cells are based on expression of tau repeat domain fragment (246–378) containing the disease-associated P301S mutation (tau-RD) fused to mCerulean3 or mClover3. The v2H line was selected for high expression with low background signal and high sensitivity. Seeding experiments used previously established protocols [14].

#### Cell culture

v2H biosensors were grown in Dulbecco’s Modified Eagle’s medium (Gibco) supplemented with 10% fetal bovine serum (HyClone), and 1% glutamax (Gibco). For terminal experiments, 1% penicillin/streptomycin (Gibco) was included. Cells were tested free of mycoplasma (VenorGem, Sigma) and cultured at 37 °C with 5% CO_2_ in a humidified incubator. To avoid false positive signal from v2H biosensors, cells were passaged prior to ~ 80% confluency.

#### Human brain samples

At the CRCHUM, fresh frozen human brain tissue was obtained from patients diagnosed with clinical and neuropathological MS according to the revised 2010 McDonald’s criteria [28]. Tissue samples were collected from MS patients with full ethical approval (BH07.001, Nagano 20.332-YP) and informed consent as approved by the local ethics committee. At UT Southwestern, human brain tissue was obtained from a 52 year old female subject with 19 year history of multiple sclerosis with Institutional Review Board approval at University of Texas Southwestern Medical Center. Informed written consent for donation of tissue was obtained from next of kin prior to collection. The brain was sectioned, tissue from each region was separated into two, with one half being flash frozen in liquid nitrogen for biochemical analysis and the other half fixed in formalin and processed to paraffin for histological analysis and immunohistochemical staining. Fresh frozen tissue was used to prepare total soluble protein lysates for further experiments.

#### Human sample preparation

Fresh frozen pulverized tissue was suspended in tris-buffered saline (TBS) containing cOmplete mini protease inhibitor tablet (Roche) at 10% w/vol. Samples were then dounce homogenized, followed by pulsing probe sonication at 75 watts for 10 min (Q700, QSonica) on ice in a hood. The sonication probe was washed with a sequence of ethanol, bleach, and distilled water to prevent cross-contamination of seeds. Lysates were then centrifuged at 23,000 × g for 30 min and the supernatant was retained as the total soluble protein lysate. Protein concentration was measured with the BCA assay (Pierce). Fractions were aliquoted and stored at − 80 °C prior to immunoprecipitation and seeding experiments.

#### Immunoprecipitation

Immunoprecipitations were performed as described previously [29]*.* 50 µL of magnetic Protein A Dynabead slurry (Thermofisher) was washed twice with immunoprecipitation (IP) wash buffer (0.05% Triton-X100 in PBS), followed by a 1 h room temperature incubation with 20 µg of anti-tau antibody. Beads were washed three times in IP wash buffer, 1000 µg of total protein lysate was added to the Protein A/anti-tau antibody complexes on the beads and rotated overnight at 4 °C. Supernatant was then removed as the tau-depleted fraction and the beads were washed three times in IP wash buffer, and then moved to clean tubes for elution. IP wash buffer was removed and beads were then incubated in 65 µL of IgG Elution Buffer (Pierce) for 7 min to elute tau. The elution buffer was collected in a separate microcentrifuge tube and a second elution step in 35 µL of IP elution buffer was performed for 5 min, and pooled with the initial elution. The tau-enriched IP pellet was then neutralized with 10 µL of Tris–HCl pH 8.4.

#### Transduction of biosensor cell lines, flow cytometry and seeding analyses

The seeding assay was conducted as previously described [14] with the following changes: v2H cells were plated 20 h before seed transduction at a density of 16,000 cells/well in a 96-well plate in a media volume of 180 µL per well. Mouse and human total protein lysates were thawed on ice, while tau-depleted IP supernatants and tau-enriched IP pellets were isolated just prior to seeding. For total protein lysates, 10 µg of protein was used per well. For tau-enriched pellets, 10 µL of elution was used per well. Samples were incubated for 30 min with 0.5 µL Lipofectamine 2000 (Invitrogen) and OptiMEM such that the total treatment volume was 20 µL. For each experiment, cells treated with OptiMEM alone and Lipofectamine 2000 in OptiMEM as negative controls. The v2H line, which expresses high levels of tau RD, can show false-positive FRET signal when treated with Lipofectamine 2000, which is mitigated by passaging prior to ~ 80% confluency. Recombinant tau fibrils at 1 pM and 100 fM (monomer equivalent) were used for positive controls. Cells were incubated for an additional 48 h after treatment prior to harvesting. Cells were harvested with 0.25% trypsin and fixed in 4% PFA for 10 min, then resuspended in flow cytometry buffer (HBSS plus 1% FBS and 1 mM EDTA). The LSRFortessa SORB (BD Biosciences) was used to perform FRET flow cytometry. Single cells double-positive for mCerulean and mClover were identified and the % FRET positive cells within this population was quantified following a gating strategy previously described [14]. For each experiment 10,000 cells were analyzed in triplicate. Flow data analysis was performed using FlowJo v10 software (Treestar).

#### Light microscopy and immunohistochemistry

Histopathologic processing, staining, and analysis were performed in the UT Southwestern Neuropathology Research Laboratory. Sections for light microscopy were cut at 4 µm on a Microm HM355S rotary microtome (ThermoScientific, Rockford, IL) and mounted on Fisherbrand Superfrost Plus positive charged slides (Fisher Scientific, Pittsburgh, PA). Adjacent sections were stained with hematoxylin and eosin, Luxol fast blue-PAS-hematoxylin, AT8 monoclonal antibody (ThermoScientific) 1:200 dilution, and MD3.1 antibody (1:16,000 dilution). AT8 and MD3.1 immunohistochemistry was performed at room temperature on a Leica Bond-III automated immunostaining platform (Leica Biosystems Inc., Buffalo Grove, IL), using the proprietary Leica Polymer Refine detection system, which includes H_2_O_2_ block, EDTA-based epitope retrieval solution, rabbit anti-mouse IgG secondary antibody, anti-rabbit poly-HRP-IgG, DAB, and hematoxylin counterstain. Stained sections were reviewed on an Eclipse NiU brightfield microscope (Nikon Instruments, Melville, NY), and virtual whole slide images for illustrations were created on an Aperio ScanScope CS2 robotic slide scanner (Leica) with 20 × objective and selected fields captured using Aperio ImageScope v.12 software.

### Statistical analyses

Fresh frozen brain regions were obtained by M.S.L. from H.M. M.S.L. remained blinded for all seeding analyses. Flow cytometry gating and analysis of seeding activity was completed prior to decoding and interpreting the results. All statistical analysis was performed using GraphPad Prism v9.2.0 for Mac OS and Excel v16.52 (Microsoft).

## Supplementary Information

**Addtional file 1: Table 1**: MS subject characteristics.

**Addtional file 2: Figure 1**: Differential epitope exposure of tau seeds in MS vs. control and AD brains. (**A**) Epitopes of antibodies used. (**B**) Differential immunoprecipitation from hippocampus of an MS brain, (**C**) parietal cortex of control brain, and (**D**) temporal cortex of AD brain. MD6.1 failed to bind seeds from MS brain, but efficiently enriched seeds from AD brain. Columns represent the mean % FRET positive cells from three technical replicates (dots).
