# Supplementary figures and images for: Tau seeding in cases of multiple sclerosis
Source: Acta Neuropathol Commun. 2022 Oct 11;10:146. doi: 10.1186/s40478-022-01444-2 (PMC9552360; doi:10.1186/s40478-022-01444-2)

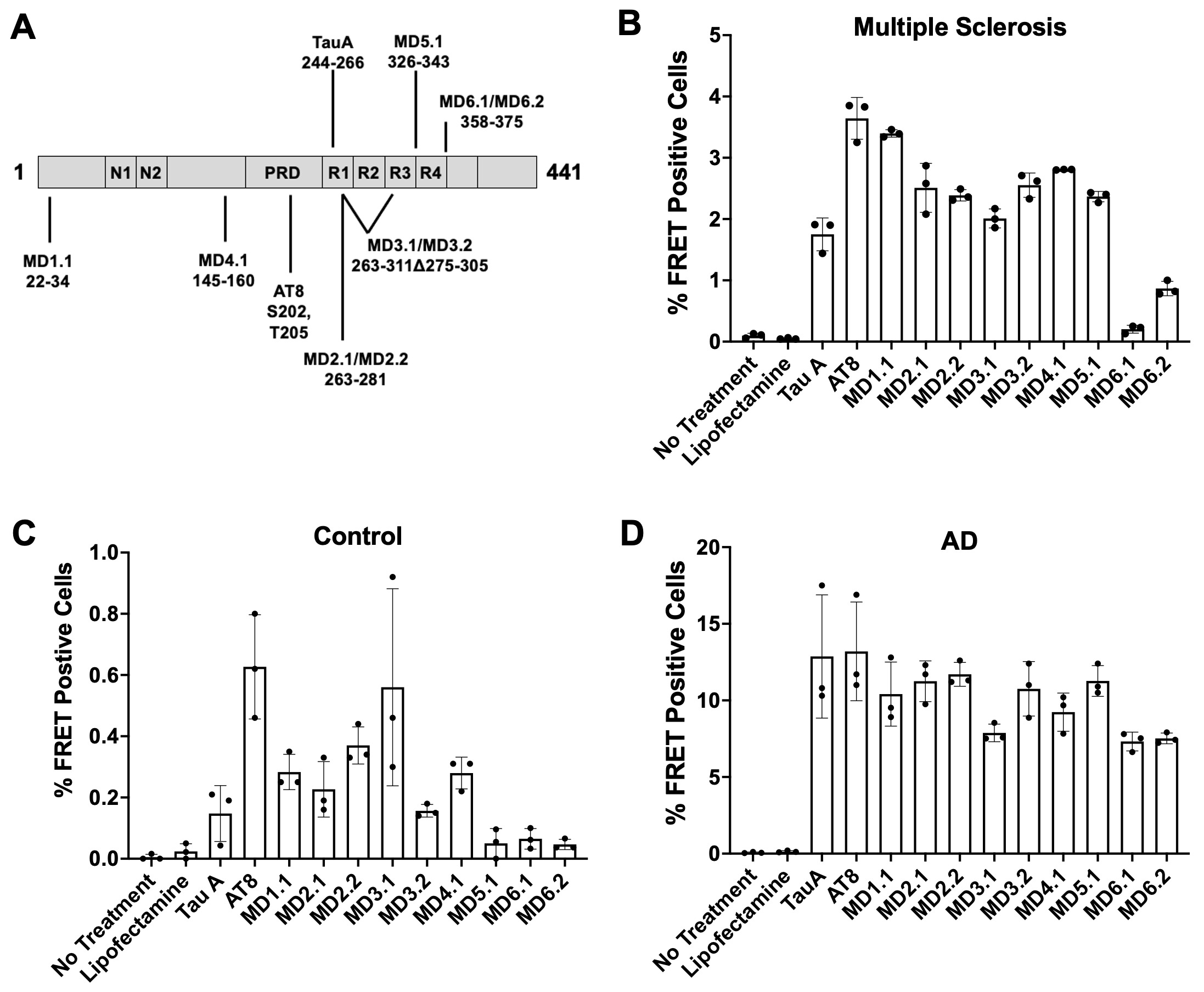

Supplement: Supplementary file 2 — Addtional file 2: Figure 1: Differential epitope exposure of tau seeds in MS vs. control and AD brains. (A) Epitopes of antibodies used. (B) Differential immunoprecipitation from hippocampus of an MS brain, (C) parietal cortex of control brain, and (D) temporal cortex of AD brain. MD6.1 failed to bind seeds from MS brain, but efficiently enriched seeds from AD brain. Columns represent the mean % FRET positive cells from three technical replicates (dots). [file 40478_2022_1444_MOESM2_ESM.png]
